# Examining Conduct Problems in a Community Sample during Middle Childhood: The Role of Frontal EEG Asymmetry, Temperament, and Working Memory

**DOI:** 10.1007/s10802-024-01191-z

**Published:** 2024-03-19

**Authors:** Mohamed Zerrouk, Martha Ann Bell

**Affiliations:** Department of Psychology, Virginia Tech, 890 Drillfield Dr., Blacksburg, VA 24060 USA

**Keywords:** EEG Frontal Asymmetry, Temperament, Executive Functioning, Middle Childhood

## Abstract

Previous literature shows that aspects of temperament, executive functioning, and EEG frontal asymmetry are related to externalizing behaviors in children. We examined whether frontal EEG asymmetry measured at age 6 would moderate the impact of negative affectivity, attentional control, and working memory at age 6 on conduct problems at age 9. Behavioral tasks were given to assess children’s attentional control and working memory. Parents completed questionnaires about their children’s negative affectivity and conduct problems. Results showed that greater negative affectivity reported at age 6 predicted for more conduct problems reported at age 9, regardless of EEG frontal asymmetry. Lower levels of attentional control and working memory at age 6 predicted for more conduct problems reported at age 9 when children also exhibited greater left EEG frontal asymmetry, which has been linked to approach motivation. These findings illustrate the importance of assessing multiple intrinsic factors, both independent and interactive, that contribute to children’s conduct problems.

Conduct problem behaviors include aggression and disobeying rules (Lopez-Romero et al., 2015). Severe levels of conduct problems may put a child at risk for developing conduct disorder (Frick & Morris, 2004), a clinical diagnosis that typically emerges in childhood or adolescence and depicted by behaviors that violate rules and the rights of others (Kazdin, 2003). Those with a childhood onset typically begin to show mild conduct problems in early childhood or middle childhood and experience growth in the amount and intensity of conduct problems throughout childhood and adolescence (Frick, 2004). For this study, we examined the impact of child factors for later conduct problems in middle childhood.

Frick and Viding (2009) suggest a developmental psychopathology approach for assessing antisocial behaviors (i.e., conduct problems, etc.) because a greater understanding of mechanisms that lead to the problem behaviors is obtained through the integration of different factors (e.g., cognitive, temperamental, etc.) and multiple level analyses. Additionally, equifinality (i.e., diverse pathways leading to the same psychopathological outcome) and multifinality (i.e., a component of a psychopathological outcome may operate differently contingent on the context) can be assessed from developmental frameworks (Cicchetti & Rogosch, 1996; Frick & Viding, 2009).

Deficits in executive functioning and self-regulation, along with temperamental and physiological risk factors, are child components related with the onset of childhood conduct problems (Dandreaux & Frick, 2009; Oosterlaan et al., 1998). Studies have shown that the interactive impact of these child factors with risk conditions external to the child (e.g., poor parenting, harsh environments, etc.) places a child at greater risk for conduct disorder (Fairchild et al., 2019; Frick, 2004). Zelazo (2020) notes that developmental approaches assess the interaction of wide-varying factors that influence psychological and biological development which can result in psychopathology. Biopsychosocial models typically focus on the interaction of a child with temperamental risk factors (e.g., high negative affectivity, poor self-regulation) and maladaptive environmental factors in the development of conduct problems (Dandreaux & Frick, 2009; Dodge & Pettit, 2003). These perspectives discuss environment as key factors interacting with child risk factors but rarely note child traits moderating other child traits. In our study, we focused on the interaction between child factors to expand our knowledge regarding child intrinsic risks for conduct problems in middle childhood.

We assessed a community sample of children at two different timepoints in middle childhood. Children at low-risk for conduct problems can experience instances of low-level aggression and disobeying, along with the potential risk that childhood problem behaviors pose for later development (Fergusson et al., 2005). Our main goal was to examine the collective impact of intrinsic child factors on the development of conduct problems prospectively in middle childhood. Our sub-goal was to examine the interactive effect of child factors, including their own neurophysiology. We focused on middle childhood because developing skills, such as EF and self-regulation, relate to the emergence (or reduction) of conduct problems during middle childhood (Austin et al., 2020).

We specifically assessed the collective impact of children’s temperamental negative affectivity, attentional control, working memory, and EEG frontal asymmetry at age 6 on children’s conduct problems at age 9. Age 6 is an important period to assess the impact of our study’s predictors because children typically begin formal education and are developing cognitive skills that allow them to plan their actions (Eccles, 1999). We focused on age 9 as an appropriate time in middle childhood to assess conduct problems because late middle childhood is a period when changes begin in the interactions children have with peers and adults (Feiring & Lewis, 1991). In addition, there is a greater predictive accuracy of conduct problems reported in later compared to earlier middle childhood (Bennet & Offord, 2001). These changes lead to a gradual disengagement from parents to children monitoring their own behavior, which can increase the likelihood of problem behavior (Dishion et al., 2004).

## Temperament and Conduct Problems

Temperament measured in early childhood is linked to subsequent child conduct problems (Lipscomb et al., 2014). Negative affectivity (NA) is a reactive component of temperament that describes the extent to which an individual experiences negative emotional states (Rothbart & Bates, 2006) and is a robust predictor of children’s conduct problems (Frick & Morris, 2004; Nigg, 2006). Deater-Deckard et al. (2007) reported that aspects of NA were related with conduct problems in a sample of twins ranging from early childhood to mid-childhood. Additionally, NA appears to increase the risk for conduct problems, as toddlers born pre-mature had a significantly greater amount of variance explained for conduct problems when having higher levels of NA (Cassiano et al., 2016). The impact of NA on conduct problems is apparent from both self-reports by children in late childhood and maternal reports (Lengua et al., 1998).

Effortful control is a self-regulatory aspect of temperament that aids children in the modulation of their attentional and emotional reactivity (Rothbart & Ahadi, 1994). Children with greater effortful control can better control their behaviors and emotions (Eisenberg et al., 2009; Liu & Bell, 2020). Attentional control (AC) is a component of effortful control and pertains to the ability to flexibly focus and shift attention (Rothbart et al., 2001). Attentional components of EC explained a significant amount of variance of twins’ conduct problems in early to middle childhood (Deater-Deckard et al., 2007). In a sample of children from age 6 through age 9, AC predicted conduct problems but only for children with lower family functioning (i.e., family cohesion and adaptability; Zeng et al., 2022). In accord with the theory and findings in the presented literature, we expected early temperament to correlate with later conduct problems during middle childhood. Specifically, we expected that higher NA and lower AC at age 6 would relate to greater conduct problems at age 9.

### Working Memory and Conduct Problems

Neurodevelopmental models highlight the importance of disrupted neural systems that support executive function skills as a factor for psychopathological symptoms (Brieant et al., 2022; Zelazo, 2020). Children with reduced executive functioning experience difficulties in learning and intentional action needed for adaptation to changing circumstances, which can contribute to conduct problems (Zelazo, 2020). We focused specifically on the executive function of working memory (WM), which is the process of maintaining and processing information in the mind during complex cognitive tasks (Baddeley, 1992). Lower WM can lead to deficits in inhibition, which in turn results in problems with impulsivity and the regulation of behavior and frustration (McQuade et al., 2013; Rapport et al., 2001). Austin et al. (2020) explain that WM deficits impact the ability to keep track of important aspects of social situations which can contribute to conduct problems. Additionally, low WM is associated with poor decision making (Bechara et al., 1998), which in turn is related to antisocial behaviors such as conduct problems (Flouri et al., 2017; Moffitt, 1993).

Research on WM impairments and conduct problems sometimes compares children with a clinical diagnosis to those without. For example, Kleine Deters et al. (2020) reported that children with clinical levels of conduct problems had impaired WM compared with healthy controls. Saarinen et al. (2015) assessed a sample of 26 boys and girls clinically diagnosed with conduct problems and 26 age and gender matched controls ranging from age 7 to age 12. Children with conduct problems had lower WM functioning compared to controls. In contrast, Flouri and colleagues (2017) showed that lower WM in a community sample of children, with varying levels of maternal depression, had an indirect, but not a direct, impact on conduct problems. In light of theory and the general findings with both clinically diagnosed and community samples, we anticipated that lower WM at age 6 will be related to greater conduct problems at age 9.

### Frontal EEG Asymmetry as Predictor and Moderator of Conduct Problems

EEG frontal asymmetry is linked with approach and withdrawal behaviors. The social approach/withdrawal model of frontal EEG asymmetry posits withdrawal behaviors are associated with right frontal activation (i.e., right frontal EEG asymmetry) and approach behaviors are associated with left frontal activation (i.e., left frontal EEG asymmetry; Davidson & Sutton, 1995). Relatively greater right frontal EEG asymmetry (FA) is linked with internalizing behaviors in early childhood (e.g., Fox et al., 2001) and relatively greater left FA is linked with externalizing behaviors in early childhood (e.g., Smith & Bell, 2010). Individuals with an overly strong approach motivation may show extremes in reward and sensation seeking and, thus, may engage in behaviors that lead to conduct problems (Nigg, 2006). Rybak et al. (2006) reported that relatively greater left FA is associated with conduct problems and suggested that a hyperactive approach system could be one neurophysiological factor putting an individual at risk for conduct problems.

There is mixed literature concerning the robustness of FA as a direct predictor of psychopathological outcomes. A meta-analysis conducted by Peltola and colleagues (2014) showed that right FA was a consistent predictor of psychosocial risk while left FA was not significant as a predictor of externalizing behaviors (Peltola et al., 2014). In a review by Reznik and Allen (2018), the authors stressed the importance of considering FA as a moderator or mediator of outcomes, rather than a direct predictor. Thus, it could be that the impact of EEG FA has differential associations with approach/withdrawal constructs depending on the context of the situation. Resting FA can be considered a physiological trait with the potential to moderate as it taps an individual difference that could promote or impede emotional responses (Coan & Allen, 2004; Reznick & Allen, 2018).

Dodge and Pettit (2003) describe an interactive model explaining that some factors may only exert risk for the development of conduct problems in the presence or absence of another risk component. This framework includes intrinsic (i.e., the individual’s traits) interactions involving physiological factors, as they note that child factors may lead to conduct problem outcomes only when they occur under biologically predisposed conditions (Dodge & Petit, 2003). Frick and Viding (2009) state multiple levels of analyses are important when examining neurological bases of cognitive and affective differences in the varying developmental pathways of antisocial behaviors (i.e., conduct problems, etc.). Aligned with the presented theoretical perspectives and research, we proposed that FA would operate as a moderator in our study.

Bates et al. (2008) note the importance of learning about the interaction of child risk factors, with a particular focus on temperament, for furthering theory of child behavior problems. Gatzke-Kopp et al. (2014) discuss an efferent model for frontal EEG asymmetry which postulates that FA moderates the relation between affective arousal and behavioral responses, such that the same emotional experience may result in different behavioral outcomes depending on the FA pattern at rest. They explain that individuals who experience greater negative affect and possess relatively greater right FA may be predisposed to internalizing symptoms while relatively greater left FA could increase the likelihood of externalizing behaviors (Gatzke-Kopp et al., 2014). This may be due to the underlying motivational tendencies of FA. Harmon-Jones and Gable (2018) explain that affective valence models depict relatively greater right EEG FA being associated with negative emotion and relatively greater left EEG FA being related to positive emotion, but that motivation may subserve these connections. Specifically, positive emotions are typically approach oriented whereas negative emotions are usually withdrawn oriented. When negative affectivity is associated with approach behaviors (e.g., conduct problems, etc.), relatively greater left FA shows greater activation, thus suggesting that the underlying motivation of affect may be related to the directionality of FA (Harmon-Jones & Gable, 2018). We have seen the interaction between temperamental affect and FA in our own work. In the same sample of children as in our current study, impulsivity-anger aspects of temperament at age 6 predicted general externalizing behavior at age 9, but only for children with relatively greater left FA at age 6 (Liu et al., 2021). Thus, we expected in our current study that relatively greater left FA would moderate the relation between NA at age 6 with conduct problems at age 9.

Bates et al. (2008) posit self-regulation differences as a potential parameter because children with patterns of low self-regulation, particularly poor AC, have a greater likelihood of developing externalizing problems when moderated by other factors. Lacey and colleagues (2020) reported that greater attentional aspects of effortful control were associated with relative right FA. Additionally, Harmon-Jones and Gable (2018) report that reductions of self-regulation post self-regulatory efforts are associated with increased approach motivation (i.e., impulsive aggression). We highlighted that lower AC is associated with conduct problems (Deater-Deckard et al., 2007; Meesters et al., 2007; Zeng et al., 2022) and FA may be more appropriate as a moderator (Coan & Allen, 2004; Reznick & Allen, 2018). Thus, we hypothesized that lesser AC at age 6 would predict for greater conduct problems at age 9 only for children that displayed relatively greater left FA. To our knowledge, this has not been examined in previous research.

A potential interaction between FA and WM is more difficult to hypothesize. It is suggested that poorer executive function may be one component that interacts with other factors across multiple levels in developmental cascades of psychopathological symptoms such as externalization (Brieant et al., 2022; Masten & Cicchetti, 2010). This would also fit with the suggestion of conducting multiple levels of analyses of neurological bases for cognitive differences in developmental pathways of externalizing behaviors (Frick & Viding, 2009). Gatzke-Kopp et al. (2012) demonstrated that highly aggressive kindergartners showed deficits in executive function, including WM, and augmented left FA during tasks that induced negative emotions. Additionally, there is evidence of individuals with internalizing symptoms showing a relation between right FA and lower executive function (Brzezicka et al., 2017). These findings along with theoretical perspectives suggest that FA may also moderate an association between executive function and risk for behavioral problems. With our focus specifically on WM, we examined whether FA moderates the relation between WM and conduct problems, to our knowledge a novel suggestion in the developmental literature. We hypothesized that lower WM at age 6 will predict for greater conduct problems at age 9 when children exhibit relatively greater left FA.

### Current Study

Determining whether FA interacts with temperament and WM to predict conduct problems will inform as to whether a neurophysiological factor, when interacting with these traits, can influence children’s problem behaviors in later childhood. Restating our objectives, we aimed to examine whether FA moderated the individual impact of NA, WM, and AC at age 6 on conduct problems at age 9. We predicted that greater NA, lesser WM, and lesser AC would respectively predict greater conduct problems at age 9 only when children display relatively greater left FA at rest at age 6.

## Method

### Participants

Participants were two cohorts of children who represented approximately 75% of a larger longitudinal study examining the development of cognition and emotion from infancy through middle childhood (i.e., the CAP Study). The 25% of the larger study not included in these analyses was a third cohort who did not have a research visit at age 6 due to the funding schedule. The two cohorts initially were recruited as infants by two research locations, a rural college town and a mid-sized city in the mid-Atlantic region. Recruitment was done using mailing lists, flyers, and word of mouth. The Blacksburg, VA research location and the Greensboro, NC research location each recruited half of the participants in the original longitudinal study.

There was the potential for 352 children to participate at age 6, based on the number of children who contributed data at the previous assessments in the longitudinal study beginning in infancy (*n* = 304) and the number of children newly recruited to join the ongoing longitudinal study at age 6 (*n* = 48). Of the 352 potential participants, there were 72 children who did not participate in the study at age 6 and 38 children who participated at age 6 via parent-report questionnaires rather than the lab visit, yielding 242 children contributing research data at age 6.

At age 9, 247 of the children had parents who completed the conduct problems measure. Thus, participants in the current study were 294 children who contributed data at either or both ages. Participants included 149 girls and 145 boys (228 White, 42 Black, 2 Asian, and 22 multiracial or other race; in addition, 20 ethnically identified as Hispanic) who were 6 years old (M = 6.64; SD = 0.44) at visit 1 and 9 years old (M = 9.30; SD = 0.37) at visit 2. 65% of mothers and 54% of fathers had a college degree. Children were healthy at the time of the age 6 and 9 visits and had no developmental delays or cognitive disabilities.

### Procedure

Data were collected at both research locations using identical protocols and identical EEG equipment. Research assistants from each location were trained together by the Principal Investigator (final author) on protocol administration, as well as data collection and psychophysiological processing. To ensure that identical protocol administration was maintained by the two research locations, the Blacksburg research group periodically viewed video recordings and provided the reliability coding for the behavioral tasks collected by the Greensboro research group. To ensure that identical EEG collection and processing were maintained by the two research locations, the Blacksburg research group periodically reviewed raw EEG files and processed EEG data collected by the Greensboro research group.

Upon arrival at the research lab for the age 6 and age 9 visits, the researcher greeted families. Consent and assent were obtained from mothers and children. Mothers sat in an adjoining room during the visit, observing through a one-way mirror and video monitor. At each visit, children performed a series of cognitive and emotional tasks not included in this study while EEG was recorded. For the current report, we used baseline EEG, behavioral attention control, behavioral working memory, and maternal-report temperament data collected at age 6, as well as maternal-report of child conduct problems at age 9. Children and parents received remuneration at both visits.

### Measures

#### Frontal EEG Asymmetry at Age 6

At the age 6 lab visit, baseline EEG was recorded for 2 min while the children sat in a chair and watched a clip of the film *Lion King* (opening scene). EEG was recorded from 16 scalp locations (international 10–20 configuration), referenced to Cz, using an Electro-Cap (Eaton, OH: E-1 series cap). After the EEG cap was placed on the head, a small amount of abrasive gel was inserted into each electrode and the scalp gently rubbed. Afterwards, a small amount of conductive gel was inserted, and electrode impedances were measured and accepted if they were below 10 K ohms.

The electrical activity from each EEG cap electrode was individually amplified with James Long Company Bioamps (James Long Company, Caroga Lake, NY). During data collection, the high pass filter was a single pole RC filter with a 0.1 Hz cutoff (3 dB or half-power point) and 6 dB per octave roll-off. The low pass filter was a two pole Butterworth type with a 100 Hz cutoff (3 dB or half-power point) and 12 dB octave roll-off. Activity for each lead was displayed on the monitor of an acquisition computer. The EEG signal was digitized at 512 Hz to eliminate the effects of aliasing. The acquisition software was Snapshot-Snapstream (HEM Data Corp., Southfield, MI). Prior to recording EEG for each child, a 10-Hz, 50 uV peak-to-peak sine wave was input through each amplifier and this calibration signal digitized for 30s. Spectral analysis of the calibration signal and computation of power at the 9 to 11 Hz frequency band was used to calibrate the power derived from the subsequent spectral analysis of the EEG.

After calibration, EEG data were examined and analyzed using the EEG Analysis software that was developed by the James Long Company. First, EEG data were re-referenced via software to an average reference configuration to eliminate concerns that power values at each active site reflect interelectrode distance as much as they reflect electrical potential. Then, average reference EEG data were artifact-scored for eye movements by using electrodes Fp1 and Fp2 to examine peak-to-peak criterion of 100 µV or greater. The EEG data also were artifact-scored for gross motor movements by using a peak-to-peak criterion of 200 µV or greater. Only artifact-free data were used in subsequent analyses. The data were then analyzed with a discrete Fourier transformation, using a Hanning window of 1-s width and 50% overlap. EEG power was expressed as mean square microvolts, and the data were transformed by using the natural log (ln) to normalize the distribution.

Power was computed for the 8–10 Hz alpha frequency band. According to research examining EEG power distribution across early development (Marshall et al., 2002), alpha corresponds to 6–9 Hz in 4-year-old children. The alpha band is typically shifted by 1–2 Hz from preschool children to school-age children (Niedermeyer, 1999). Therefore, alpha likely corresponds to 8–10 Hz in 6-year-old children. This frequency band has been used in FA research with children in the middle and late childhood age range (Forbes et al., 2005). We focused on FA using electrode locations F3 and F4, which have been consistently associated with emotion, motivation, and behavioral problems (Reznik & Allen, 2018). The value for FA was calculated by subtracting the natural log-transformed power at the left hemisphere (F3) from the natural log-transformed power at the right hemisphere (F4). Because cortical activity is inversely related to alpha power (Reznik & Allen, 2018), left FA is indicated by positive EEG asymmetry values, which means greater left relative to right brain activation. Right FA is indicated by negative EEG asymmetry values, which means a greater right to left brain activation.

### Attentional Control at Age 6

Using a modified version of the NEPSY subtest (Espy & Bull, 2005), children were presented with an 8 × 14 inch laminated page containing a photograph of small colorful items. They were asked to point only to items that matched the target item (i.e., stars) on the page containing both distractors and targets. Trained research assistants later coded the task using the recording of the laboratory visit. A unique efficiency score was calculated as the proportion of target responses (i.e., 9 stars) that were touched only one time. Reliability coding for this task was accomplished on 20% of the sample. The intraclass correlation (ICC) between coders was 0.978.

### Working Memory at Age 6

Working memory at age 6 was measured by the backward digit span task. Children were read a seemingly random series of single-digit numbers (0–9) and then attempted to reproduce the sequence in reverse. A practice trial with two sets of two digits was given to acclimate to the task. Each trial in the task contained two different sets of digits of the same amount. The task began with a set of two-digit sequences and then added one more digit in each subsequent trial. Children had two chances to correctly reproduce the new digit sequence in reverse for each set. The task ended when the child provided incorrect responses on both chances for each set in a trial. The last correct trial was used as the child’s backward digit span score.

### IQ at Age 6

The Peabody Picture Vocabulary Test (PPVT–IV; Dunn & Dunn, 2007) was administered to children and used as a proxy for verbal intelligence. The PPVT–IV is a nationally standardized instrument and the measure of interest was participants’ age equivalence based on a standardized score.

### Negative Affect at Age 6

Temperamental negative affect at age 6 was measured with the Children’s Behavior Questionnaire-Short Form (CBQ-SF; Putnam & Rothbart, 2006). Mother reported her child’s behaviors on a 7-point Likert scale ranging from 1 (*extremely untrue of your child*) to 7 (*extremely true of your child*). This 95-item questionnaire assessed the child’s emotional and behavioral responses across different situations. Negative affect is a factor formed from the CBQ-SF scales for fear, sadness, anger/frustration, discomfort, and falling reactivity/soothability (reverse scored). Cronbach’s alpha for the negative affect factor was 0.838.

### Conduct Problems at Age 9

The Child Behavior Checklist (CBCL 6–18; Achenbach et al., 2001) is a 118-item questionnaire that was used to measure parent observations of children’s emotional and social problems on a 3-point Likert scale from 0 (*not true*) to 2 (*very/often true*). The focus of this study was the conduct problems scale (12 items) at age 9. We used the raw scale score rather than T-scores because of our community sample, with higher scores indicating greater levels of conduct problems. Cronbach’s alpha for the conduct problems scale was 0.795.

### Missing Values

Of the 294 children in this study, 196 had data for all variables. Expectation-maximization (EM) in SPSS 28 was used to account for the missing values of the variables in our regression analyses. Across all variables, the average percentage of missing values was 16.9% (6.1% for age 6 NA, 18.7% for age 6 FA, 18.0% for age 6 AC, 19.0% for age 6 WM, 19.0% for age 6 IQ, and 16.0% for age 9 conduct problems). For the age 6 variables, 14 participants did not contribute any data to the age 6 visit and 38 contributed only questionnaire data to the age 6 visit. Thus, 52 participants had no behavioral data at age 6. In addition, the IQ, AC, and WM tasks were not administered to one, one, and four children, respectively, at age 6; three children were missing FA data (two did not wear cap, one equipment failure); and four parents did not return the CBQ, so there was no NA data for those children. For the age 9 conduct problems measure, 46 participants did not return for the age 9 visit and therefore had no data for the CBCL questionnaire. One participant who did contribute data for the age 9 visit had no data for the CBCL questionnaire.

Other techniques for dealing with missing data (e.g., listwise deletion and pairwise deletion) can introduce biased estimates when the proportion of missing data is above 5% or data is not missing completely at random (MCAR; Dong & Peng, 2013). EM specifically accounts for the conditions under which the missing data occurred, thus providing better estimates for parameters than either listwise deletion or pairwise deletion (Dong & Peng, 2013). The calculated MCAR from the EM analysis was not significant (*χ*^2^ = 40.630, *df* = 36, *p* = .274) indicating that the data were missing at random. Additionally, the EM dataset (*n* = 294) and observed dataset (using listwise deletion, *n* = 196) were separately analyzed to assess the results and observe any evidence of bias that may have resulted from the implementation of EM. The descriptive statistics and results were similar between the two respective datasets, justifying the results from EM. Thus, we presented the results from the EM dataset.

## Results

All analyses were conducted using SPSS 28. Descriptive statistics and correlations are shown in Table 1. Values that were 3 standard deviations from the mean were considered outliers. There were 1 outlier (above the mean) for FA, 1 outlier (above the mean) for NA, 1 outlier (above the mean) for WM, and 9 outliers (all above the mean) for conduct problems. Outliers were winsorized.

**Table 1:** Descriptive Statistics and Correlations for Age 6 Negative Affect, Working Memory, Attentional Control, and Frontal EEG Asymmetry with Age 9 Conduct Problems

	N	Mean	SD	Range	1	2	3	4	5	6	7	8
1. NA	276	3.94	0.75	1.93–6.15	---							
2. AC	241	0.564	0.27	0.00–1.00	− 0.06	---						
3. FA	239	0.014	0.16	-0.45-0.52	− 0.05	0.009	---					
4. WM	238	3.02	0.81	1.00-4.50	− 0.08	0.004	− 0.04	---				
5.Conduct Problems	247	1.52	2.2	0.00–9.00	0.23^*^	− 0.15^*^	0.02	− 0.12	---			
6. IQ	241	7.83	1.3	4.75–11.9	0.01	0.05	− 0.17^*^	0.35^*^	− 0.20^*^	---		
7. MED	294	4.87	1.1	2.00–7.00	− 0.07	− 0.04	− 0.10	0.19^*^	− 0.24^*^	− 0.37^*^	---	
8. PED	294	4.71	1.5	1.00–7.00	− 0.12^*^	− 0.005	− 0.09	0.25^*^	− 0.27^*^	− 0.48^*^	0.59^*^	---

Note. Values after winsorizing. NA = negative affect; AC = attentional control; FA = frontal EEG asymmetry, WM = working memory, IQ = intelligence as assessed by PPVT, MED = maternal education, PED = paternal education

*p < .05

A three-step hierarchical regression model was conducted to examine whether NA, AC, and WM at age 6 respectively predicted conduct problems at age 9 and whether FA at age 6 interacted with all three predictors; NA, AC, and WM respectively in predicting conduct problems (see Table 2). Step 1 included child sex, child IQ, maternal education level, and paternal level education as covariates. Lower levels of child IQ and parent socioeconomic status are related to externalizing problems (Hinshaw, 1992) and boys greatly outnumber girls in having conduct problems (Moffitt & Caspi, 2001). Step 1 explained 10.3% of the variance. Step 2 added age 6 NA, AC, WM, and FA as predictors to the model. The change in R^2^ for step 2 (*p* < .001) accounted for an additional 11.2% of the variance in child conduct problems at age 9.

**Table 2:** Hierarchical Regression Analysis of Age 6 Child NA, Age 6 AC, Age 6 Child FA, and Age 6 WM Predicting Age 9 Child Conduct Problems

		B	Std. Error	Beta	CI	
Step 1						
	Sex	− 0.556	0.202	− 0.154^**^	− 0.952, − 0.159	
	Mom edu	− 0.076	0.116	− 0.046	− 0.305, 0.152	
	Dad edu	− 0.245	0.090	− 0.197^**^	− 0.423, − 0.067	
	IQ	− 0.141	0.089	− 0.098	− 0.316, 0.033	
	R^2^ = 0.103, F (4, 289) = 8.341, *p* < .001					
Step 2						
	Sex	− 0.624	0.191	− 0.173^**^	− 0.999, − 0.249	
	Mom edu	− 0.089	0.110	− 0.053	− 0.305, 0.126	
	Dad edu	− 0.175	0.086	− 0.141^*^	− 0.345, − 0.005	
	IQ	− 0.173	0.090	− 0.120	− 0.349, 0.003	
	NA	0.614	0.137	0.245^***^	0.345, 0.884	
	AC	-1.44	0.383	− 0.199^***^	-2.20, − 0.687	
	WM	− 0.163	0.140	− 0.065	− 0.439, 0.113	
	FA	− 0.058	0.658	− 0.005	-1.35, 1.24	
	*R*^*2*^*change* = 0.112, F (4, 285) = 10.162, *p* < .001					
Step 3						
	Sex	− 0.580	0.189	− 0.161^**^	− 0.952, − 0.209	
	Mom edu	− 0.046	0.109	− 0.028	− 0.260, 0.168	
	Dad edu	− 0.173	0.085	− 0.139^*^	− 0.340, − 0.005	
	IQ	− 0.172	0.088	− 0.119	− 0.346, 0.003	
	NA	0.539	0.138	0.215^***^	0.268, 0.801	
	AC	-1.37	0.381	− 0.188^***^	-2.12, − 0.617	
	WM	− 0.150	0.139	− 0.060	− 0.423, 0.123	
	FA	− 0.354	0.656	− 0.029	-1.65, 0.937	
	NA * FA	− 0.219	0.940	0.013	-2.07, 1.63	
	AC * FA	-5.706	2.796	− 0.109^*^	-11.2, − 0.202	
	WM * FA	-2.876	0.968	− 0.166^**^	-4.87, − 0.971	
	*R*^*2*^*change* = 0.034, F (3, 282) = 4.225, *p* = .006					

Note. CI = 95% confidence intervals; NA = negative affect; AC = attentional control; FA = frontal EEG asymmetry; WM = working memory; Mom edu = mother education; Dad edu = father education; IQ = intelligence as assessed by PPVT.

*p < .05

**p < .01

***p < .001

Step 3 added the interactions between FA with NA, AC, and WM. The variables included in the interaction terms were centered before being included in the regression equation. The change in R^2^ for step 3 (*p* = .006) accounted for an additional 3.4% of the variance in child conduct problems. NA was significant as a main effect (*b* = 0.215, *p* < .001), with the positive beta weight indicating that greater levels of NA at age 6 predicted greater conduct problems at age 9. AC was significant as a main effect (*b* = − 0.188, *p* < .001), with the negative beta weight indicating that lower levels of AC at age 6 predicted greater conduct problems at age 9.

**Fig. 1:**
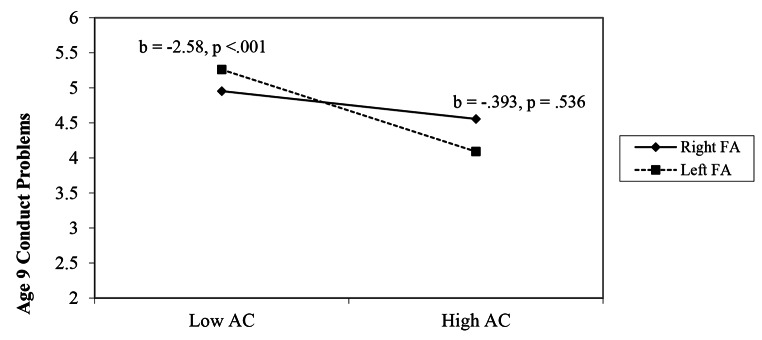
Age 6 frontal EEG asymmetry moderates the relation between Age 6 attentional control and age 9 conduct problems. *Note.* AC = attentional control. FA = frontal EEG asymmetry; left FA are positive numbers indicative of relatively greater left FA and right FA are negative numbers indicative of relatively greater right FA

**Fig. 2:**
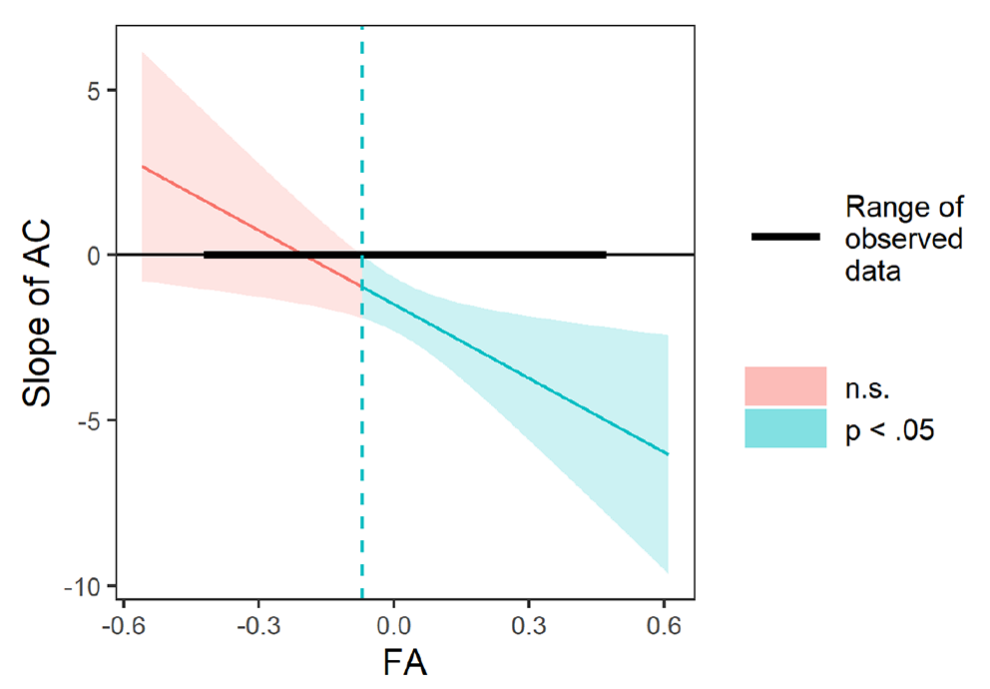
Johnson-Neyman plot of age 6 child AC interacting with age 6 child EEG frontal asymmetry in predicting the conduct problems at age 9. Note. AC = attentional control and FA = EEG frontal asymmetry. The vertical line represents the boundary of significance, whereby the effect of AC on conduct problems was significant only for children with AC values to the right of the vertical line (-0.071)

The interaction between AC and FA was significant (*b* = − 0.109, *p* = .042). Using PROCESS 3.5 (Hayes, 2017), simple slopes analyses were conducted to examine interaction effects. As shown in Fig. 1, AC at age 6 was negatively associated with conduct problems at age 9 among children with relatively greater FA at age 6 (1 SD above the mean; i.e., relatively greater left FA; simple slope *b* = -2.58, *p* < .001), but not among children with relatively lower FA at age 6 (1 SD below the mean; i.e., relatively greater right FA; *b* = − 0.393 *p* = .536). A Johnson-Neyman plot (Fig. 2) shows the breadth of the interaction indicating that as FA gradually moves leftward from the point of significance (-0.071), the negative slope, specifically less AC and more conduct problems, is significant.

**Fig. 3:**
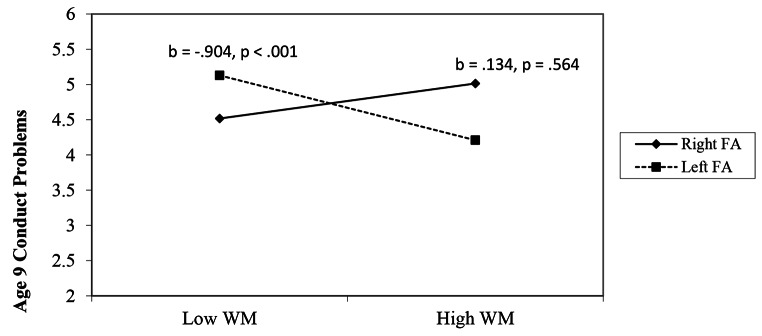
Age 6 frontal EEG asymmetry moderates the relation between age 6 working memory and age 9 conduct problems. *Note.* WM = working memory. FA = frontal EEG asymmetry; left FA are positive numbers indicative of relatively greater left FA and right FA are negative numbers indicative of relatively greater right FA

**Fig. 4:**
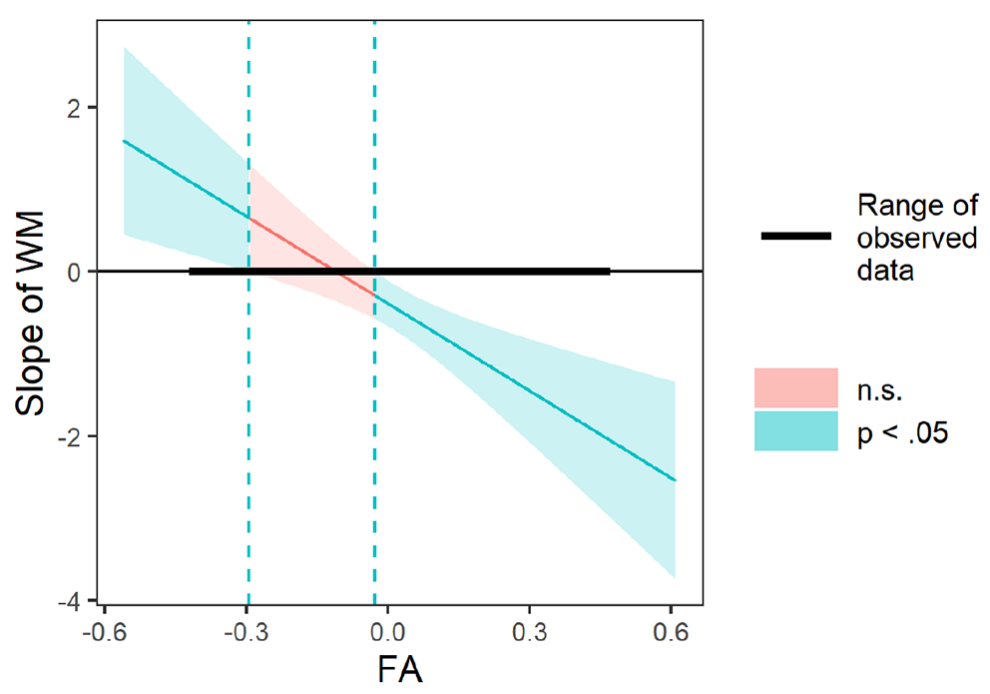
Johnson-Neyman plot of age 6 child WM interacting with age 6 child EEG Frontal Asymmetry in Predicting the Conduct Problems at Age 9. Note. WM = working memory and FA = EEG frontal asymmetry. The vertical line represents the boundary of significance, whereby the effect of WM on conduct problems was significant for children with positive WM values to the left of the first vertical line (FA = − 0.295) and with negative WM values to the right of the second vertical line (FA = − 0.028)

Additionally, the interaction between WM and FA was significant (*b* = − 0.166, *p* = .003). Simple slopes analyses were conducted. As shown in Fig. 3, WM at age 6 was negatively associated with child conduct problems at age 9 among children with relatively greater FA at age 6 (1 SD above the mean; i.e., relatively greater left FA; *b* = − 0.904, *p* < .001), but not among children with relatively lower FA at age 6 (1 SD below the mean; i.e., relatively greater right FA; *b* = 0.134, *p* = .564). The Johnson-Neyman plot (Fig. 4) shows the breadth of the interaction between the negative slope of WM and conduct problems. As FA gradually increases (i.e., greater relative left FA) from one point of significance (-0.028), lesser WM and greater conduct problems are significant. At the second point of significance (-0.295), as participants’ FA gradually decreases (i.e., greater relative right FA), greater WM and lesser conduct problems are significant.

## Discussion

We assessed interactions between FA and aspects of temperament, as well as WM, in predicting conduct problems in a community sample. Our first hypothesis was partially supported. Greater levels of NA at age 6 predicted for higher number of conduct problems reported at age 9. Previous studies have shown that NA is associated with conduct problems (Cassiano et al., 2016; Lengua et al., 1998; Nigg, 2006), as well as generalized externalizing problems during middle childhood (Eisenberg et al., 2009). The other aspect of our first hypothesis, that relatively greater left FA at age 6 would moderate the relation between NA reported at age 6 and age 9 conduct problems reported was not supported. This finding warrants further examination, as left FA is associated with approach behaviors such as conduct problems (Rybak et al., 2006). However, meta-analyses of findings regarding FA and general externalization suggest differently. Peltola and colleagues (2014) reported evidence via a meta-analysis that relatively greater left FA did not significantly predict for externalizing behaviors while right FA consistently predicted for psychosocial risk.

Researchers have long proposed that FA should be considered in analyses as a moderator or as a mediator because resting FA has the potential to moderate individual differences in the elicitation or inhibition of emotional responses (Coan & Allen, 2004; Reznick & Allen, 2018). This view along with developmental psychopathological perspectives (Dandreaux & Frick, 2009; Dodge & Pettit, 2003; Masten & Cicchetti, 2010) motivated us to examine the interactive effect of FA on NA and on our other main predictors (i.e., AC and WM). There are plausible reasons, however, why FA did not moderate the relation between NA at age 6 and conduct problems at age 9 in our sample.

First, it could be simply that NA is a robust enough predictor for externalizing behaviors, and specifically conduct problems in this study, that neurophysiological individual differences will not alter the association. Thus, regardless of a child’s FA, children with greater NA will predict greater conduct problems. Second, it could be that only specific components of NA, and not NA wholistically, interact with left FA in predicting greater conduct problems. This second potential reason seems to be more likely when taking into consideration a study which had the same sample as this study (Liu et al., 2021). A composite variable derived from two aspects of NA, anger and impulsivity, at age 6 predicted for externalizing behaviors at age 9, as did the interaction between anger/impulsivity and FA. Thirdly, NA and conduct problems were reported by the same rater (i.e., maternal report). Same rater reports can induce bias covariance which may inflate the effect size between variables (Hoyt, 2000). Thus, the sole reliance on maternal reports for measuring children’s conduct problems at age 9 is a limitation. A meta-analysis by De Los Reyes and colleagues (2015) indicates moderate agreement (*r* = .30) between maternal and teacher reports of externalizing symptoms in children, noting the importance of multi-informant assessment for more valid accounts of behavior problems. Mothers may rate their children as having more externalizing behaviors compared to teachers (Miner & Clarke-Stewart, 2008; Santos et al., 2020). Therefore, having mother rate both NA and conduct problems may contribute to a bias resulting in a stronger association between the constructs. Lastly, Pérez-Edgar and Hastings (2018) note questionnaire reports measuring NA are limited in capturing affect in the moment or context of risk. This suggests behaviorally measuring NA in conditions triggering risk of externalizing problems could be of benefit. Future studies should examine both NA and individual components of NA interacting with FA in predicting conduct problems and then use a composite of parent and teacher report along with behavioral measures of NA in risky contexts to reduce bias in assessing whether there are differential results as present in this study. Lastly, it is unlikely that FA has no relation with conduct problems because FA was significant as a moderator in our second and third hypotheses respectively.

Our second hypothesis, that FA at age 6 would moderate the relation between age 6 AC and age 9 conduct problems was supported. Specifically, our result indicates that children with lower scores of AC predict greater conduct problems reported at age 9 only when displaying relatively greater left FA. To our knowledge, this is a novel finding. The interaction between lesser AC and left FA adds support to Bates et al. (2008) suggestion of examining differences in self-regulation as an important component for the development of externalization, especially in moderation analyses. Even without including the interaction of FA with AC, previous studies have suggested that lower AC is positively associated with conduct problems in children (Deater-Deckard et al., 2007; Meesters et al., 2007; Zeng et al., 2022). Future studies can build on this result by assessing whether FA also moderates the relation between lower AC and conduct problems in (a) earlier development (i.e., early childhood) and (b) in later development (i.e., adolescence), or whether the moderation is specific to middle childhood.

Our last hypothesis, that FA at age 6 would moderate the relation between age 6 WM and age 9 conduct problems was supported. Specifically, children with lower scores of WM predicted greater conduct problems reported at age 9, but only when displaying relatively greater left FA. This finding to our knowledge is also novel. The most similar result we found was a longitudinal study that indicated WM was a moderator, in which children with poor spatial WM exacerbated the impact of maternal depression on child conduct problems across ages 3, 5, 7, and 11 (Flouri et al., 2017). The result of our study indicates the interactive potential of WM by evidencing the negative impact of lower WM with a neural disposition (i.e., FA) as a moderator on conduct problems. Additionally, this result supports developmental perspectives regarding the potential of lower executive functioning capabilities interacting with other risk factors in the onset of externalization (Brieant et al., 2022; Masten & Cicchetti, 2010; Frick & Viding, 2009). Future studies should attempt to replicate left FA interacting with WM in predicting conduct problems in later childhood. Similarly, it is important for future research to examine whether FA moderates the relation between lower WM and greater conduct problems in other times of childhood, such as early childhood and adolescence.

The finding that relatively greater left FA moderates the impact of lower WM and AC respectively in predicting greater conduct problems adds greater weight to the suggestion by Reznik and Allen (2018) that the impact of FA is better assessed as a moderator (or mediator) on outcome variables. Thus, it could be that relatively greater left FA does not predict externalizing behaviors as a main effect, and in this case conduct problems, but only when interacting with other important traits in children. Secondly, these results may support the notion that FA is an underlying index of motivation for affect which can predispose children to the risk of developing later psychopathological symptoms, in this case greater relative left FA with behaviors associated with externalizing problems. Thirdly, our finding suggests that relatively greater left FA is a risk factor that further aggravates the impact that lower AC and WM respectively have on conduct problems in middle childhood. Lastly, our result may add valuable information to equifinality perspectives (Cicchetti & Rogosch, 1996) related to conduct problems because we demonstrated that interactions among intrinsic child risk factors can predict the same outcome (i.e., conduct problems) as interactions between child and environmental risk components.

We note that our findings should be replicated in both early childhood and adolescence because these are other developmentally sensitive periods in which children are at risk for the development of conduct disorder (Frick, 2004). Thus, it is important to understand whether relatively greater left FA is an interactive risk factor for temperament (i.e., AC) and executive function (i.e., WM), only in the early-middle childhood onset group or also a risk factor for other age onset groups as well. These future findings may present more detail as to what can influence the development of conduct problems in children. Additionally, children that showed relatively greater right FA with low AC and WM at age 6 respectively did not significantly predict greater conduct problems at age 9. Results also indicate that greater relative right FA moderates the relation between higher WM and lower conduct problems. This may suggest that relatively lesser left FA is a protective factor against conduct problems for children with lower AC or WM in the early years of middle childhood. Thus, future research should assess the protective benefits of relatively lesser left FA asymmetry as well.

Although our findings suggest relatively greater left FA can serve as a risk factor, our results also indicate that FA alone does not predict conduct problems. Additionally, our results suggest that FA could be a multifinality factor because the potency of risk can change contingent on the context. Specifically, whether left or right FA is shown, if FA is a moderator or not, and the risk components that interact with FA determine if FA significantly increases conduct problems or not. Therefore, it may be pertinent to focus on early deficits in WM and AC because our correlations showed children with higher WM and higher AC had lower conduct problems reported. Early intervention may stunt the trajectory of childhood conduct disorder onset and prevent severe symptoms from manifesting later in development (Pardini & Frick, 2013). Although interventions need to target multiple risk factors in the development of the childhood onset of conduct disorder, it is crucial to have individualized programs that efficaciously focus on specific factors (Pardini & Frick, 2013). Research has shown that WM can be enhanced via targeted training tasks in non-clinical samples of children (Morrison & Chein, 2011). Additionally, AC training premised on mindfulness (Chambers et al., 2008) and cognitive tasks (Wass et al., 2011) in non-clinical samples have been related to improvement in AC. Lastly, it has been suggested that programs focusing on enriching cognitive skills in early childhood may act as a preventive measure of later externalizing behaviors (Liu, 2004). Future studies could assess whether lower WM and AC at an earlier timepoint in childhood is associated with conduct problems at a later timepoint after children receive training on improving AC and WM capabilities. Such findings would provide evidence of additional ways to help prevent the development of conduct disorder in late childhood and potentially hinder a cascade of behavioral problems prevailing into adolescence and adulthood. Research continuing to discover unique factors contributing to the behavior problems of children with conduct problems may aid in the development of both comprehensive and individualized programs targeting prevention and treatment (Pardini & Frick, 2013).

It is also important to note that our study was composed of a non-clinical sample of children when considering the ramifications of these findings. Although we used a clinical assessment, the DSM scale of conduct problems on the CBCL (Achenbach et al., 2001), these children were not diagnosed as having conduct disorder. Importantly, the level of conduct problems in our sample was generally low. Our results could suggest that even in a sample of children with low severity of conduct problems, visible effects of potential risk factors can still be observed. However, these strong effects may not be seen in clinical samples, such that other important factors may better explain the risks associated with conduct problems. Nevertheless, future research should assess whether the findings of our study generalize to clinical samples, especially those with diagnosed conduct disorder. It is important to assess the impact of early middle childhood factors on late middle childhood conduct problems because middle and late childhood conduct problems are associated with adulthood crime and substance use (Fergusson et al., 2005) and is a critical period for the development of diagnosed conduct disorder (Frick, 2004).

Another consideration of this study is the sole focus on conduct problems without assessing the impact of other externalizing behaviors that are often in comorbidity with conduct problems, such as oppositional defiant disorder (ODD), attention-deficit/hyperactivity disorder (ADHD), and callous-unemotional (CU) traits (Fanti, 2013; Hudec & Mikami, 2018). Research indicates that symptoms from these externalizing behaviors can overlap with one another in child and adolescent community samples (Andershed et al., 2018; Truedsson et al., 2019). Future research can control for ODD, ADHD, and CU traits to examine whether there are cognitive and physiological processes that are uniquely linked with conduct problems. By understanding developmental vulnerabilities that are specific to conduct problems, practitioners may be able to focus on treatments for combating these distinct risk factors for children displaying high levels of conduct problems in middle childhood.

Lastly, the use of multiple sites to collect data can be considered. By doing so, our study was able to recruit a larger, more diverse sample. However, a potential limitation for some multi-site studies may be that different situations at the sites influence results. This is more threatening in observational studies predicated on interpreting actions due to the risk of contrasting perspectives or interpretations in coding variables used in subsequent analyses (Whitley & Kite, 2012). Because our study used quantitative measures (i.e., Likert-scale questionnaires, physiological indices, and rule-based behavioral assessments), it is less likely to be a concern for this study. However, future research may be additionally cautious by controlling site location in studies where differential interpretations of variables is a potential concern.

In conclusion, our study demonstrated the interactive impact that the neurophysiological marker FA has with AC and WM in predicting conduct problems. Our data suggest that interventions focused on deficits in AC and WM, especially for children with left FA, may be an effective approach in the treatment of children experiencing conduct problems in late middle childhood.
